# Late Effects and Survivorship Issues in Patients with Neuroblastoma

**DOI:** 10.3390/children5080107

**Published:** 2018-08-06

**Authors:** Danielle Novetsky Friedman, Tara O. Henderson

**Affiliations:** 1 Department of Pediatrics, Memorial Sloan Kettering Cancer Center, 1275 York Avenue, New York, NY 10065, USA; 2 Department of Pediatrics, Weill Cornell Medical College, New York, NY 10065, USA; 3 Department of Pediatrics, Comer Children’s Hospital, University of Chicago, Chicago, IL 60637, USA; thenderson@peds.bsd.uchicago.edu

**Keywords:** neuroblastoma, late effects, childhood cancer, survivorship

## Abstract

Over the past two decades, marked progress has been made in understanding the biology of neuroblastoma; this has led to refined risk stratification and treatment modifications with resultant increasing 5-year survival rates for children with neuroblastoma. Survivors, however, remain at risk for a wide variety of potential treatment-related complications, or “late effects”, which may lead to excess morbidity and premature mortality in this cohort. This review summarizes the existing survivorship literature on long-term health outcomes for survivors of neuroblastoma, focusing specifically on potential injury to the endocrine, sensory, cardiovascular, pulmonary, and renal systems, as well as survivors’ treatment-related risk for subsequent neoplasms and impaired quality of life. Additional work is needed to assess the potential late effects of newer multimodality therapies with the aim of optimizing long-term medical and psychosocial outcomes for all survivors of neuroblastoma.

## 1. Introduction

Neuroblastoma is the most common extracranial solid tumor of childhood, with approximately 800 cases diagnosed per year in the United States, typically in children under the age of five [1]. The disease is marked by its clinical heterogeneity, with five-year survival rates ranging from 95% for those with low-risk disease to 50% for those with high risk disease [2]. With recognition of this discrepancy, therapeutic efforts over the past several decades have focused on de-escalating therapy for those with low or intermediate risk disease, and intensifying therapy for those with high-risk disease [3,4,5,6,7,8,9,10,11]. Thus, contemporary treatment for low-risk disease is often restricted to surgery alone with limited use of chemotherapy [12], while patients with intermediate risk disease receive multiple cycles of chemotherapy with surgery (and radiation, if needed), and those with high-risk disease receive multimodality therapy including high-dose chemotherapy, radiotherapy, stem cell transplantation, and novel biologic and immunotherapeutic approaches, including *cis*-retinoic acid [9,13,14,15,16,17].

Given the wide variation in stage-based treatment protocols, survivors of neuroblastoma are at risk for a broad variety of treatment-related morbidities, or “late effects”. These complications are directly related to the types of therapies administered as well as demographic factors such as age at treatment, sex, and genetic background [18,19,20,21,22,23]. Given the complexity of these patients, continued lifelong care in a dedicated long-term follow-up program is ideal. However, this review provides an overview of the most common late effects and survivorship issues for individuals with a history of neuroblastoma and is intended for the pediatrician who may be providing continued care to survivors of neuroblastoma. Systematic monitoring of these potential late toxicities allows for early detection, treatment, and prevention of long-term morbidity in this population, with the aim of improving the long-term quantity and quality of life of survivors.

## 2. Late Effects in Survivors of Childhood Cancer: An Overview

The largest reports of long-term morbidity and mortality in neuroblastoma survivors have emerged from the North American Childhood Cancer Survivor Study (CCSS), a multi-institutional retrospective cohort study that provides longitudinal follow-up on 5-year survivors of a range of childhood cancers diagnosed before the age 21 years [24,25]. In a landmark study of 10,937 childhood cancer survivors, which included individuals diagnosed with neuroblastoma, leukemia, central nervous system tumors, Hodgkin’s lymphoma, non-Hodgkin’s lymphoma, Wilms’ tumor, soft-tissue sarcoma, and bone tumors between 1970 and 1986, the cumulative incidence of any chronic health condition at 30 years after primary cancer diagnosis was 73.4%, with an estimated incidence of 42.4% for a severe, disabling, life-threatening, or fatal condition [26]. While neuroblastoma-specific risk or stage data were not included in this analysis, it is likely, given the dates of diagnosis, that the majority of included neuroblastoma survivors had low- or intermediate-risk disease.

Since that initial report, treatment has intensified for high-risk neuroblastoma patients, with resultant increases in therapy-related toxicity. It is thus not surprising that in a recent analysis on changing trends in late mortality among CCSS childhood cancer survivors diagnosed from 1970–1999, neuroblastoma survivors were one of the only diagnostic sub-groups found to have an increase in late mortality among those treated in more recent decades [27]. We hypothesize that the cohort of patients treated in the 1990s includes a higher proportion of high-risk patients, relative to survivors diagnosed in the 1970s and 1980s, which is associated with both improved survival and an increased burden of late toxicities (due to treatment with novel combinations of intensive multimodality therapy).

## 3. Long-Term Medical Outcomes in Survivors of Neuroblastoma

Several analyses have focused specifically on the delineation of late effects in neuroblastoma survivors. One analysis of 954 5-year CCSS neuroblastoma survivors, diagnosed between 1970 and 1986, found that survivors of neuroblastoma had an increased rate of mortality and second malignancies, when compared to age- and sex-matched US population controls, and an 8-fold increased risk of chronic health conditions, when compared to 3899 CCSS siblings [22]. The most prevalent chronic medical conditions involved the neurological, sensory, endocrine, and musculoskeletal systems. Survivors treated with multimodality therapy were 2.2-fold more likely to develop a chronic health condition, when compared to survivors treated with surgery alone. It is likely that most of these patients had low- or intermediate-risk disease, which was potentially over-treated.

With the introduction of risk stratification and intensification of therapy for those with high-risk disease, recent analyses have focused on long-term toxicities among this subset of survivors [18,20,21,28,29]. One single-institution study of 63 advanced stage neuroblastoma survivors, the majority of whom were treated with multimodality therapy between 1970 and 2001, detected late complications in 95% of survivors, including hearing loss (62%), primary hypothyroidism (24%), ovarian failure (41% of females), musculoskeletal abnormalities (19%), and pulmonary abnormalities (19%) [21]. Other reports have similarly observed a high prevalence of late effects in this cohort, the majority of which are related to the endocrine system, ototoxicity, or subsequent malignancies [20,21,23,30,31,32].

While many of these complications are mild–moderate in severity, a recent report of long-term risk of hospitalization in survivors of childhood cancer in Denmark, Finland, Iceland, and Sweden found that survivors of neuroblastoma were at highest risk among all diagnostic groups for subsequent hospitalization [33]. Risk for diseases requiring inpatient treatment occurred many decades after cancer therapy, thus underscoring the need for continued risk-based screening and long-term follow-up for both mild–moderate and severe complications among survivors of neuroblastoma. Table 1 outlines select neuroblastoma-directed therapies and their potential late effects. 

### 3.1. Endocrinopathies

Endocrinopathies are one of the most prevalent complications after treatment of childhood cancer [34,35] and are especially common among high-risk neuroblastoma survivors treated with multimodality therapy [20,21,36,37]. In one report of 25 long-term high-risk neuroblastoma survivors treated with high-dose chemotherapy with autologous stem cell transplant without the use of total body irradiation (TBI), the majority of patients had endocrine-related late effects, including primary hypothyroidism, growth failure, and hypogonadism [18].

#### 3.1.1. Thyroid Dysfunction

Survivors exposed to radiation to the neck, such as TBI, and/or high-dose alkylating agents, particularly when administered prior to transplant, are at risk for developing primary hypothyroidism [35,38]. High rates of irreversible thyroid dysfunction have been reported after iodine-131 metaiodobenzylguanidine (I-131 MIBG) therapy [39,40,41]. Survivors with these risk factors should have annual monitoring of thyroid stimulating hormone (TSH) levels, with serum free T4 levels added in those with elevated TSH levels. Treatment with levothyroxine is indicated for any survivor diagnosed with primary hypothyroidism.

#### 3.1.2. Linear Growth and Bone Health

Poor linear growth is common among neuroblastoma survivors, particularly among those exposed to conditioning regimens containing TBI [42,43,44]. One study of 51 high-risk neuroblastoma survivors (*n* = 41 exposed to TBI) found that height was significantly impacted, with a change in Z-score of −1.91 for those exposed to TBI and −0.77 for those not exposed to TBI [20]. Survivors exposed to TBI or cranial radiation (with hypothalamic-pituitary doses ≥18 Gy) may be at risk for growth hormone deficiency and central precocious puberty [45]. As mentioned above, neuroblastoma survivors are also at high risk for thyroid dysfunction [20,41], which can negatively impact linear growth if not adequately treated [36].

In addition to these endocrine etiologies of poor growth, neuroblastoma survivors’ final height may be negatively impacted by non-endocrine causes as well, such as radiation-induced direct damage to the growth plates of the spine or long bones [46]. Single-institution reports have also indicated that those exposed to 13-*cis*-retinoic acid may be at risk for advanced bone age and premature epiphyseal fusion [47], which may further affect survivors’ final height [47]. 

Radiation to bone, which is known to have a direct cytotoxic effect on epiphyseal chondrocytes, can also lead to localized osteopenia and kyphoscoliosis [48]. Individuals with therapy-related growth hormone deficiency or hypogonadism are also at risk for bone mineral density deficits [48]. Survivors with low bone mineral density should be counseled about dietary changes and supplementation, when indicated, to ensure adequate calcium and vitamin D intake.

All survivors with suboptimal linear growth should be referred to an endocrinologist for consideration of growth hormone stimulation testing. Those with kyphoscoliosis identified on physical exam should be referred to an orthopedist for consideration of further management.

#### 3.1.3. Hypogonadism and Impaired Fertility

Childhood cancer survivors treated with intensive multimodality therapy, including those with a history of neuroblastoma, are at risk for gonadal dysfunction. Females treated with high-dose alkylating agents prior to autologous stem cell transplant and/or TBI may experience premature ovarian insufficiency and/or future infertility [49,50,51]; primary ovarian insufficiency has also been reported in females treated with I-131 MIBG therapy [52]. Given their young age at treatment, female neuroblastoma survivors treated with intensive multimodality therapy may experience absent, arrested, or delayed puberty and require referral to a pediatric endocrinologist for appropriate management [53]. Males treated with high-doses of alkylating agents and/or TBI are at risk for future infertility as well [54,55,56].

At entry to survivorship, risk for future infertility and alternative family building options should be discussed with all survivors in a developmentally appropriate way that respects the patient’s personal values. For female survivors who experience menarche spontaneously with normal gonadotropins, post-treatment fertility preservation via oocyte or embryo freezing is available. Those interested should be referred to a reproductive endocrinologist for further discussion, evaluation, and management.

#### 3.1.4. Diabetes and Metabolic Syndrome

Neuroblastoma survivors treated with abdominal radiation are at increased for components of the metabolic syndrome [57,58] and diabetes mellitus [59,60]. When compared to siblings, survivors of neuroblastoma exposed to abdominal radiation had a 6.9-fold increased risk for diabetes; neuroblastoma survivors without a history of abdominal radiation were not at increased risk [59]. Individuals treated with TBI are at even higher risk for these complications [20,59,61,62,63]. Further complicating this risk for metabolic dysfunction, male neuroblastoma survivors have been found to be less physically active than their peers [64]. It is thus imperative for survivors of neuroblastoma exposed to abdominal radiation or TBI to be counseled regularly about the importance of dietary modification and regular physical activity as a preventive measure. At-risk survivors should be monitored with a fasting blood glucose or hemoglobin A1c every two years. Those with elevated blood glucose levels should be referred to a pediatric endocrinologist for further management.

### 3.2. Ototoxicity

Given the wide use of platinum-based chemotherapy for the treatment of neuroblastoma, profound hearing loss is a pervasive problem, particularly among those with a history of high-risk disease [29,65,66,67,68,69,70,71]. The prevalence of ototoxicity in this cohort ranges from 13% to 95% in published reports; exposure to cisplatin in combination with myeloablative doses of carboplatin significantly increases risk [32]. Exposure to ototoxic antibiotics during therapy likely compounds this risk. Neuroblastoma survivors with hearing loss have been found to be at increased risk for learning problems and psychosocial impairments [72]. In order to optimize educational and psychosocial outcomes in this cohort, it is essential for neuroblastoma survivors to be screened for ototoxicity and referred for hearing services and hearing aids, if indicated, in a timely manner. A recent report has also indicated that neuroblastoma survivors with profound hearing loss may be good candidates for cochlear implantation [73], which may be a therapeutic option for some affected survivors. Furthermore, early phase studies of instillation of agents into the middle ear to reduce platinum-related ototoxicity are underway.

### 3.3. Pulmonary Dysfunction

Adverse pulmonary outcomes have been reported as a late effect of neuroblastoma therapy [74], particularly among those with high-risk disease [21,75,76], and are thought to be multifactorial in nature. One study of 39 high-risk neuroblastoma survivors who underwent pulmonary function testing (PFT) found that 33% reported chronic respiratory symptoms; however, PFT abnormalities were mostly mild to moderate in severity [77]. Another case series reported on six survivors of high-risk neuroblastoma who developed bronchiectasis; all six patients were treated with multimodality therapy including busulfan/melphalan prior to autologous transplant [78], which is known to induce pulmonary toxicity.

Individuals exposed to known therapies with potential pulmonary toxicity (radiation to the lungs, pulmonary-toxic chemotherapies) should have a baseline PFT at entry to long-term follow-up, with testing repeated as clinically indicated. Patient should be counseled about smoking avoidance as well.

### 3.4. Cardiac Dysfunction

Cardiovascular disease contributes to early morbidity and mortality among all childhood cancer survivors treated with cardiotoxic therapy, such as anthracyclines and/or chest-directed radiation, and may include cardiomyopathy/heart failure, coronary artery disease, stroke, pericardial disease, arrhythmias, and valvular dysfunction [79,80]. Given that doxorubicin is an integral component of many protocols for the treatment of neuroblastoma patients in North America (albeit with lower cumulative doses than other childhood cancer survivor cohorts), survivors should receive appropriate risk-based screening with serial echocardiograms [81] and report any new cardiac symptoms to their treating physician. With regards to neuroblastoma specifically, one study showed that survivors of neuroblastoma had a 4.1-fold increased risk of congestive heart failure, 11.1-fold increased risk of myocardial infarction; 5.1-fold increased risk of pericardial disease; and 7.7-fold increased risk of valvular abnormalities, when compared to unaffected siblings [82], thus highlighting the need for careful cardiac follow-up in this cohort and the need for vigilant reporting of symptoms to a medical provider. All survivors should be screened with echocardiograms at intervals tailored to their cumulative dose of anthracyclines and/or history of chest-directed radiation exposure [81].

### 3.5. Renal Dysfunction

The data on renal dysfunction among neuroblastoma survivors are conflicting. A recent Cochrane review of early and late renal dysfunction in childhood cancer survivors treated with nephrotoxic therapies, including cisplatin, carboplatin, ifosfamide, radiation therapy involving the kidney region, and/or nephrectomy, found that the prevalence of renal adverse effects ranged from 0% to 84% across studies [83]. Definitive conclusions could not be drawn regarding the prevalence of renal dysfunction, or its risk factors, after administration of these potentially nephrotoxic therapies. However, a recent single-institution analysis of 266 high-risk neuroblastoma survivors treated with abdominal radiation found that zero patients developed chronic renal insufficiency at a median follow-up of 3.5 years [84]. Given that neuroblastoma survivors often undergo nephrectomy, receive potentially nephrotoxic chemotherapy, radiation to the kidney(s), and potentially nephrotoxic antibiotics, further work is needed to clarify survivors’ risk for late renal toxicity.

### 3.6. Subsequent Malignant Neoplasms and Second Neoplasms

Neuroblastoma survivors treated with contemporary intensive multimodality therapies are at increased risk for subsequent malignant neoplasms (SMNs), including treatment-related acute myelogenous leukemia (AML) [23,30,85,86]. A review of 5987 patients in the International Neuroblastoma Risk Group database demonstrated that the 10-year cumulative incidence of a SMN among high-risk patients was 1.8%, compared with 0.38% for low-risk patients (*p* = 0.01) [30]. In this study, the standardized incidence ratio (SIR) of AML, or the ratio of the observed numbers of patients with AML compared to the expected numbers in the general population, was 106.8 and 127.7, respectively, for patients treated on high- and intermediate-risk clinical trials [30]. Others have similarly reported a high incidence of secondary AML/myelodysplastic syndrome (MDS) [87,88] as well as increased risk of secondary AML/MDS with increase in the number of dose-intensive chemotherapy cycles received [89].

In addition to hematologic malignancies, neuroblastoma survivors are also at risk for a variety of subsequent solid SMNs. It is well known that prior radiation exposure is associated with the development of subsequent solid tumors in all cancer survivors [90,91], including those with a history of neuroblastoma [92]. Additionally, a specific association has been noted between neuroblastoma and secondary renal cell carcinoma (RCC) [93,94,95,96,97], which may be related to prior cisplatin exposure [97]. Among 954 neuroblastoma survivors in the CCSS original cohort, eight patients developed RCC with an SIR of 85.8; neuroblastoma survivors had the highest risk of secondary RCC among all primary diagnostic groups [97,98]. Neuroblastoma survivors have also been found to have increased SIRs for subsequent head and neck carcinomas (SIR 20.9) and female genitourinary carcinomas (SIR 19.1) [98], secondary thyroid papillary carcinoma, chondrosarcoma, hepatocellular carcinoma, biliary adenocarcinoma [87], and melanoma [99]. In order to improve SMN-related morbidity and mortality in this cohort, it is imperative that survivors follow recommended SMN screening guidelines [81], including early breast cancer screening for females exposed to chest-directed radiation, colorectal cancer screening for those exposed to high-dose radiation to the abdomen, and skin cancer screening for individuals previously exposed to therapeutic radiation. 

Survivors of neuroblastoma have also been shown to be at risk for benign subsequent neoplasms, including osteochondromas or osteocartilaginous exostoses, both inside and outside the radiation field [100], and hepatic focal nodular hyperplasia [101,102,103,104], which may be incidentally noted on routine surveillance imaging. Survivors and their family members should be reassured about the benign nature of these lesions when detected incidentally on routine imaging or clinical examination. 

## 4. Long-Term Psychosocial and Psychological Outcomes

In addition to potential medical morbidities, neuroblastoma survivors have been shown to be at increased risk for psychological problems and impaired quality of life. A recent study of 859 5-year survivors of neuroblastoma diagnosed between 1970 and 1999 with attained age <18 years showed that survivors had an increased prevalence of impairment in the domains of anxiety/depression, headstrong behavior, attention deficits, peer conflict/social withdrawal, and antisocial behavior (16% vs. 12%; *p =*  0.01), when compared to 872 siblings [105]. Psychological impairment was associated with special education service usage and educational attainment less than college [105]. Interestingly, there was no association between treatment intensity and worse psychological outcomes in this cohort. While increased risk of psychological distress has been noted when comparing neuroblastoma survivors to siblings, no differences were noted when comparing individuals with a history of neuroblastoma to other solid tumor survivors [106]. 

Prior analyses have similarly shown that neuroblastoma survivors score below the population mean score on the Mental Component Summary of the Short Form-36 (SF-36) (population mean = 50; neuroblastoma mean = 42.4; *p* <  0.0001), thus reflecting decreased emotional health [107]. Another study showed that adult neuroblastoma survivors had lower individual and household incomes; were less likely to have ever been employed; and were less likely to have ever married, when compared to siblings, thus suggesting decreased social integration [22]. It is unclear whether more contemporarily treated neuroblastoma survivors experience similar psychosocial and health-related quality of life outcomes; further work is needed to clarify quality of life and psychosocial outcomes in this cohort.

Given their young age at treatment, however, it is reasonable to refer neuroblastoma survivors for formal neuropsychological testing at entry to long-term follow-up, in order to enable implementation of educational supports at a young age and optimize educational achievement in this cohort.

## 5. Risk-Based Screening and the Importance of Survivorship Care

Given the high prevalence of late medical and psychosocial morbidities in neuroblastoma survivors, and the unique long-term needs of this population, it is imperative that survivors of neuroblastoma receive appropriate lifelong risk-based care in a dedicated survivorship clinic. Many of the toxicities described have a prolonged, clinically silent latency period; appropriate risk-based screening can enable early detection and treatment, when possible, of potential treatment-related complications.

In the United States, survivorship clinics generally follow the evidence-based surveillance guidelines published by the Children’s Oncology Group (COG), which are updated every 5 years and publicly available online at http://www.survivorshipguidelines.org [81]. Additionally, the Children’s Oncology Group is currently recruiting participants for the LEAHRN (Late Effects After High-Risk Neuroblastoma) Study, which will comprehensively assess late effects in 5-year survivors of high-risk neuroblastoma diagnosed on or after 1 January 2000. 

Efforts are also underway to harmonize guidelines internationally between international cooperative groups, including COG, the Dutch Childhood Oncology group (DCOG), the UK Children’s Cancer and Leukaemia Group (CCLG), and the Scottish Intercollegiate Guidelines Network (SIGN) [108]. Once complete, this effort will establish an integrated strategy for the surveillance of late effects in childhood and young adult cancer survivors worldwide. 

**Figure 1:**
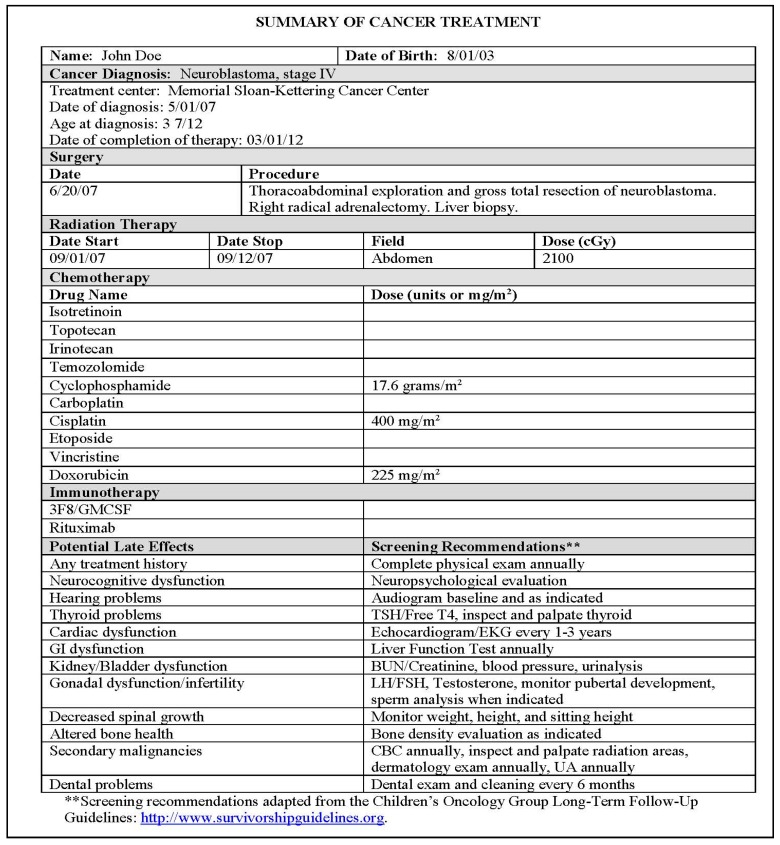
Sample treatment summary for a survivor of neuroblastoma. Abbreviations: cGy, centigray; GM-CSF, Granulocyte-Macrophage Colony Stimulating Factor; TSH, thyroid stimulating hormone; free T4, free thyroxine; EKG, electrocardiogram; BUN, blood urea nitrogen; LH, luteinizing hormone; FSH, follicle stimulating hormone; CBC, complete blood count; UA, urinalysis. 3F8 is a murine monoclonal IgG3 antibody specific for the tumor- associated antigen ganglioside GD2, which is used at Memorial Sloan Kettering Cancer Center.

At our institutions, at entry to long-term follow-pp, survivors and their families receive a summary of all cancer treatments, or survivorship care plan, received with an outline of potential late effects and COG-based screening recommendations (Figure 1). In addition to a comprehensive history and physical examination, appropriate screening tests are coordinated on the day of the visit. We also review the importance of healthy lifestyle behaviors, such as diet and exercise, as well as the dangers of cigarette smoking and secondhand smoke, and review academic performance and psychosocial health. All patients have the option of meeting with a dedicated nutritionist and/or social worker at each survivorship visit. Depending on the intensity of treatment received, and the risk for late effects, survivors are generally followed every 6 or 12 months for continued risk-based screening and management of potential complications that arise. Ideally, all survivors should be followed by a primary care physician in conjunction with the treating cancer center (either the primary oncologist and/or a dedicated long-term follow-up program) and follow risk-based screening as outlined on the survivorship care plan. Lifelong annual survivorship visits, which are generally covered by insurance, are recommended for early detection and management of potential late effects.

## 6. Conclusions

Neuroblastoma survivors face lifelong medical and psychosocial risks related to their prior cancer therapies. The most prevalent complications in this cohort include conditions involving hearing, the endocrine system, and SMN, although survivors are at risk for disturbances in other systems as well, as outlined above. In order to promote early identification and treatment of these problems, lifelong surveillance and risk-based follow-up care should be provided for all survivors, particularly those treated with intensive multimodality therapy. The goal of survivorship care for this cohort is to reduce morbidity and mortality, educate and empower both survivors and their families, and enhance quality of life among all neuroblastoma survivors. 

## Figures and Tables

**Table 1:** Potential late effects after select neuroblastoma-directed therapies.

Potential Late Effect	Therapeutic Exposure
Thyroid dysfunction	Radiation to the neck or scatter
	Total body irradiation
	Iodine-131 Meta-iodobenzylguanidine (I-131 MIBG) therapy
	High-dose alkylating agents prior to transplant
Growth hormone deficiency	Radiation to the hypothalamic-pituitary axis (≥18 Gy)
	Total body irradiation
Gonadal dysfunction	Alkylating agents
	Cisplatin
	Radiation to the gonads
Skeletal dysplasia	Radiation to the spine or long bones
	Cis-retinoic-acid
Diabetes Mellitus	Abdominal radiation
	Total body irradiation
Hearing loss	Cisplatin
	Myeloablative doses of carboplatin
	Ototoxic antibiotic exposures
Pulmonary dysfunction	Busulfan
	Radiation to the chest or upper abdomen
Cardiac dysfunction	Anthracyclines
	Radiation to the chest or upper abdomen
Renal dysfunction	Nephrectomy
	Platinum-based chemotherapy (cisplatin, carboplatin)
	Radiation therapy involving the kidney
Subsequent malignancies	Epipodophyllotoxins
	Alkylating agents
	Anthracyclines
	Radiation therapy
